# Net Clinical Benefit of Non-vitamin K Antagonist Oral Anticoagulants for Venous Thromboembolism Prophylaxis in Patients With Cancer: A Systematic Review and Trade-Off Analysis From 9 Randomized Controlled Trials

**DOI:** 10.3389/fphar.2018.00575

**Published:** 2018-06-12

**Authors:** Yi-Dan Yan, Chi Zhang, Long Shen, Ying-Jie Su, Xiao-Yan Liu, Li-Wei Wang, Zhi-Chun Gu

**Affiliations:** ^1^Department of Pharmacy, Renji Hospital, School of Medicine, Shanghai Jiaotong University, Shanghai, China; ^2^Department of Cardiology, Renji Hospital, School of Medicine, Shanghai Jiaotong University, Shanghai, China; ^3^Department of Oncology, State Key Laboratory for Oncogenes and Related Genes, Renji Hospital, School of Medicine, Shanghai Jiaotong University, Shanghai Cancer Institute, Shanghai, China

**Keywords:** non-vitamin K antagonist oral anticoagulants, dabigatran, rivaroxaban, apixaban, edoxaban, cancer, venous thromboembolism, net clinical benefit

## Abstract

Venous thromboembolism (VTE) is highly prevalent in patients with cancer. Non-vitamin K antagonist oral anticoagulants (NOACs), directly targeting the enzymatic activity of thrombin or factor Xa, have been shown to be as effective as and safer than traditional anticoagulation for VTE prophylaxis in no-cancer patients. However, related studies that focused on the anticoagulation in cancer patients are lacked, and almost no net clinical benefit (NCB) analyses that quantified both VTE events and bleeding events have been addressed in this fragile population. Therefore, we aim to investigate this issue using a systematic review and NCB analysis. A comprehensive search of Medline, Embase, and Cochrane Library were performed for randomized controlled trials (RCTs) that reported the VTE events and major bleeding of NOACs and traditional anticoagulants in patients with or without cancer. Odds ratios (ORs) and 95% confidence intervals (CIs) of VTE and bleeding events were calculated using a random-effects model. The primacy outcome of narrow NCB was calculated by pooling ORs of VTE and major bleeding, with a weighting of 1.0. Similarly, the broad NCB was calculated by pooling ORs of VTE and clinically relevant bleeding. Heterogeneity was assessed through *I*^2^ test and Q statistic, and subgroup analyses were performed on the basis of different patients (VTE patients or acutely ill patients), comparators (vitamin-K antagonists or low-molecular-weight heparin), and follow-up duration (≤6 months or >6 months). Overall, 9 RCTs including 41,454 patients were enrolled, of which 2,902 (7%) were cancer patients, and 38,552 (93%) were no-cancer patients; 20,712 (50%) were administrated with NOACs and 20,742 (50%) were administrated with traditional anticoagulants. The use of NOACs had a superior NCB than traditional anticoagulation in both cancer patients (OR: 0.68, 95%CI: 0.50-0.85 for narrow NCB; OR: 0.76, 95%CI: 0.61–0.91 for broad NCB) and no-cancer patients (OR: 0.75, 95%CI: 0.54-0.96 for narrow NCB; OR: 0.85, 95%CI: 0.67–1.04 for broad NCB), with the estimates mainly from VTE patients receiving long-term warfarin treatment. In conclusion, NOACs may represent a better NCB property compared to traditional anticoagulants in cancer patients who need long-term anticoagulation treatment.

## Introduction

Venous thromboembolism (VTE) is highly prevalent in patients with cancer, occurring up to 15% of cancer patients during the course of their diseases (Caine et al., 2002; Elalamy et al., 2017). A prominent role is attributed to ability of tumor cells to destabilize the coagulation system including releasing procoagulant proteases, expressing tissue factor on cancer cells, deriving microvesicles, as well as altering the extracellular matrix of the cancer cell milieu (Nickel et al., 2016). These patients, when receiving anticoagulant treatment, have a high risk of recurrent VTE, while prossessing the danger of bleeding complications (Prandoni et al., 2002). Therefore, the optimal anticoagulant strategy for banlancing VTE and bleeding poses a major challenge in this fragile population. Lee AYY et al conducted the CLOT trial comparing dalteparin with warfarin for preventing recurrent VTE in 672 patients with cancer, and revealed that low-molecular-weight heparin (LMWH) was more effective than vitamin-K antagonists (VKAs) in reducing the risk of recurrent VTE, without increasing the major bleeding risk (Lee et al., 2003). LMWH thus is the recommended anticoagulant for the treatment of cancer-associated VTE.

Of late years, non-vitamin K antagonist oral anticoagulants (NOACs), with a predictable dose response and no need for laboratory monitoring, have been shown to be as effective as and probably safer than conventional anticoagulation when regarding VTE treatment and prophylaxis (Schulman et al., 2009, 2013, 2014; Bauersachs et al., 2010; Goldhaber et al., 2011; Büller et al., 2012, 2013; Agnelli et al., 2013; Cohen et al., 2013; Raskob et al., 2018), which makes these agents more appealing for long-term VTE prevention. Whereas, clinical trials of NOACs that specially aimed at patients with cancer are lacked, and only a minor proportion of cancer patients (<5%) was involved in NOACs studies (Schulman et al., 2009, 2014; Bauersachs et al., 2010; Goldhaber et al., 2011; Büller et al., 2012, 2013; Agnelli et al., 2013; Cohen et al., 2013; Raskob et al., 2018). For a meaningful analysis, several meta-analysis studies have been conducted on the focus of this issue by pooling those 5% patients, and demonstrated that the use of NOACs seem to be as effective and safe as warfarin/LMWH for the VTE prophylaxis in cancer patients (Larsen et al., 2014; Van Der Hulle et al., 2014; Vedovati et al., 2015; Brunetti et al., 2017; Di Minno et al., 2017). However, insufficient sample size was the main limition for aboved meta-analysis studies. Encouragingly, the latest Hokusai-Cancer trial involving large sample size of 1,050 patients with predominantly advanced cancer and acute symptomatic or incidental VTE have been published in Feb 2018, which met the sample size requirement for estimating a reduction in VTE from 3 to 5% (Raskob et al., 2018). In addition, the concept of net clinical benefit (NCB) has been growingly used to quantify both thromboembolism and hemorrhage in the field of anticoagulant treatment, while no NCB studies to date have been specifically addressed in NOACs-treated patients with cancer. Hence, it is necessary to address this important knowledge gap by using a trade-off analysis from previous randomized controlled trials (RCTs) as well as recently published Hokusai-Cancer trial.

## Methods

### Data sources and searches

The present systematic review was conducted in accordance with standards outlined in the Cochrane Handbook and the PRISMA Statement for Reporting Systemic Reviews and was performed according to the priori established protocol (PROSPERO: CRD42018089939). We searched Medline, Embase, and Cochrane Library electronic databases to identify all potential eligible trials from inception to Feb 14th, 2018, with the following searching strategy: “dabigatran” or “Pradaxa” or “rivaroxaban” or “Xarelto” or “apixaban” or “Eliquis” or “edoxaban” or “Savaysa” or “Betrixaban” or “Bevyxxa” or “Non-vitamin K antagonist oral anticoagulants” or “NOACs” or “direct oral anticoagulants” or “DOACs” or “novel oral anticoagulants” or “new oral anticoagulants” or “factor Xa inhibitors” or “factor IIa inhibitors” AND “venous thromboembolism” or “VTE” or “pulmonary embolism” or “PE” or “deep vein thrombosis” or “DVT” AND “clinical trial” or “controlled clinical trial” or “randomized controlled trials.” References of all pertinent articles were further scrutinized to ensure that all relevant studies were identified. Two reviewers (Yi-Dan Yan and Chi Zhang) independently searched the databases, and all disagreements were resolved by consulting a third author (Zhi-Chun Gu).

### Study selection and outcomes

The primacy outcomes were narrow NCB weighting both VTE events and major bleeding events (MBEs), as well as broad NCB balancing VTE events and clinically relevant bleeding events (CRBEs: MBEs and clinically relevant non-major bleeding events). Studies were considered potentially eligible for this systematic review if they met the following predetermined criteria: (1) only RCTs that reported intested data of patients with or without cancer were included; and (2) VTE events, MBEs, or CRBEs were objectively assessed in NOACs groups and traditional anticoagulation groups, respectively. For duplicate publications of the same RCTs, the most relevent to our inclusion criteria was considered. When regarding possibly selective bias, two reviewers (Yi-Dan Yan and Chi Zhang) were blinded to authors' names, journal names, and publication years of the papers, and all disagreements were resolved through discussion and the opinion of a third reviewer (Zhi-Chun Gu).

### Data extraction and quality evaluation

All data that followed intention-to-treat principle were extracted independently by two reviewers (Yi-Dan Yan and Chi Zhang) using a priori designed form that included number of patients, mean age, sex, mean weight, body mass index (BMI), type of VTE, duration of follow-up, VTE events, MBEs, CRBEs in patients with or without cancer. The methodological quality of trials was evaluated based on the Cochrane Collaboration Risk of Bias Tool, which included random sequence generation, allocation concealment, blinding, incomplete outcome data, selective reporting, and other bias (Higgins et al., 2011; Wei et al., 2016). Potential publication bias was evaluated by visually inspecting funnel plots if more than 10 studies were included (Gu et al., 2017).

### Data analysis

Odds ratios (ORs) and their 95% confidence intervals (CIs) of VTE events and bleeding events were calculated by employing a random-effects model. The narrow NCB of NOACs vs. traditional anticoagulation was calculated by pooling ORs of VTE and MBEs, with a weighting of 1.0. Similarly, the broad NCB was conducted by merging ORs of VTE and CRBEs, with a weighting of 1.0. The overall estimates were presented in forest plots, and weighting of each study was assigned on the basis of event rate and sample size. Heterogeneity, defined as variation beyond chance, was evaluated through the *I*^2^ test and Q statistic that measures the percentage of total variation between studies. *I*^2^ of >50% indicated considerable heterogeneity, and a *p* < 0.05 at Q statistic represented a significant heterogeneity (Higgins et al., 2003). Subgroup analyses of NCB were calculated by different patients (VTE patients or acutely ill patients), comparisons (vitamin-K antagonists or low-molecular-weight heparin), and duration of follow-up (≤6 months or >6 months). Sensitivity analysis was also performed for detecting the effect of a single trial by sequential elimination of each trial from the pool, and afterward to reassess the overall effects. All statistical analyses were performed by using STATA software (version13, Statacorp, College Station, Texas, USA), and *P* < 0.05 indicated a statistically significant difference.

## Results

### Study evaluation

**Figure 1:**
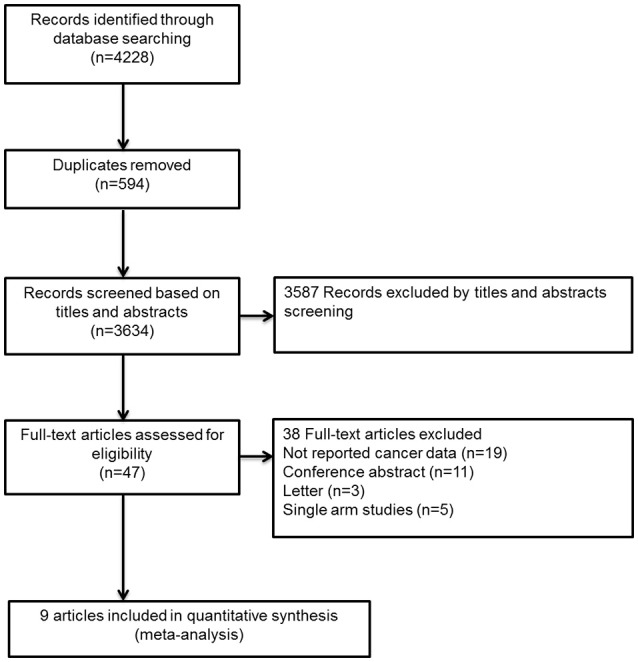
Flow diagram for the selection of eligible randomized controlled trials.

The flow diagram for study selection was shown in Figure 1. The literature search yielded 4,228 records, of which 47 full-text articles were obtained to further assess for eligibility, and 9 eligible RCTs were included in the final analyses (Schulman et al., 2009, 2014; Bauersachs et al., 2010; Goldhaber et al., 2011; Büller et al., 2012, 2013; Agnelli et al., 2013; Cohen et al., 2013; Raskob et al., 2018). The characteristics and defination of outcomes in included RCTs were presented in Table 1 and Supplemental Table 1. A total of 41,454 patients were enrolled, of which 2,902 were cancer patients (1,499 patients exposed to NOACs and 1,403 patients exposed to traditional anticoagulation) and 38,552 were no-cancer patients (19,213 patients allocated to NOACs and 19,339 patients allocated to traditional anticoagulation). Of these 9 studies, 7 studies concerned about patients with VTE and 2 studies concerned about acutely ill patients. The median age of patients ranged from 54 to 71 years and the percentage of male ranged from 40 to 60. Also, The duration of follow-up ranged from 1 to 12 months across the 9 trials. All trials satisfied bias tool items with the exception of EINSTEIN trial and Hokusai-Cancer trial, which were open-label studies. Thus, the included studies had low bias overall, meaning that the quality of the included trials was very high (Table 2).

**Table 1:** Summarized characteristics of included randomized controlled trials.

**Source**	**Patients**	**Drugs**	**N**	**Mean age (y)**	**Male (%)**	**Mean weight**	**BMI (kg/m^2^)**	**CCr30-50 (%)**	**DVT (%)**	**Follow up(month)**
AMPLIFY	Cancer	Apixaban	88	65.5	56.8	79.16	NA	8	67	6
		Enoxaparin/warfarin	81	65.1	60.5	81.69	NA	13.6	88	
	No Cancer	Apixaban	2417	56	59.1	84.88	NA	5.2	66	
		Enoxaparin/warfarin	2444	55.6	59.5	84.84	NA	4.7	69	
EINSTEIN-DVT/PE	Cancer	Rivaroxaban	258	NA	59	NA	27.4	13^*^	NA	12
		Enoxaparin/warfarin	204	NA	53	NA	26.7	17^*^	NA	
	No Cancer	Rivaroxaban	3563	NA	55	NA	28.2	7^*^	NA	
		Enoxaparin/warfarin	3594	NA	54	NA	28.2	7^*^	NA	
Hokusai	Cancer	Edoxaban	109	67	50	NA	NA	NA	58	12
		Warfarin	99	66	61	NA	NA	NA	59	
RE-COVER-I/II	Cancer	Dabigatran	114	63.5	49	78.1	27.6	NA	75	6
		Warfarin	107	65.3	45	76.1	26.8	NA	74	
	No Cancer	Dabigatran	2380	54.3	40.1	84.7	28.7	NA	69	
		Warfarin	2392	54	40.6	84	28.5	NA	68	
Hokusai-Cancer	Cancer	Edoxaban	522	64.3	53.1	78.8	NA	7.3	37	12
		LMWH	524	63.7	50.2	79.1	NA	6.5	37	
MAGELLAN	All	Rivaroxaban	4050	71	55.6	77.5	28.2	21.5	NA	1
		LMWH	4050	71	52.7	77.3	28.2	21.5	NA	
ADOPT	All	Apixaban	3255	66.8	50	NA	NA	NA	NA	1
		LMWH	3273	66.7	48.2	NA	NA	NA	NA	

BMI, body mass index; CCr, creatinine clearance rate; DVT, deep vein thrombosis; NA, not available; N, number of patients;

* *EINSTEIN-DVT/PE study provided combined data of patients whose CCr < 50 ml/min*.

**Table 2:** Quality assessment.

**Study**	**Random sequence generation**	**Allocation concealment**	**Blinding of participants and personnel**	**Blinding of outcome assessment**	**Incomplete outcome data**	**Selective reporting**	**Other bias**
AMPLIFY, 2013	L	L	L	L	L	L	L
EINSTEIN-DVT, 2010	L	U	H	L	L	L	L
EINSTEIN-PE, 2012	L	U	H	L	L	L	L
Hokusai-VTE, 2013	L	U	L	L	L	L	L
RE-COVER I, 2009	L	L	L	L	L	L	L
RE-COVER II, 2014	L	U	L	L	L	L	L
Hokusai-Cancer, 2017	L	U	H	L	L	L	L
MAGELLAN, 2013	L	U	L	L	L	L	L
ADOPT, 2011	L	U	L	L	L	L	L

*L, low risk; U, unclear risk; H, high risk*.

### NCB analysis in patients with and without cancer

**Figure 2:**
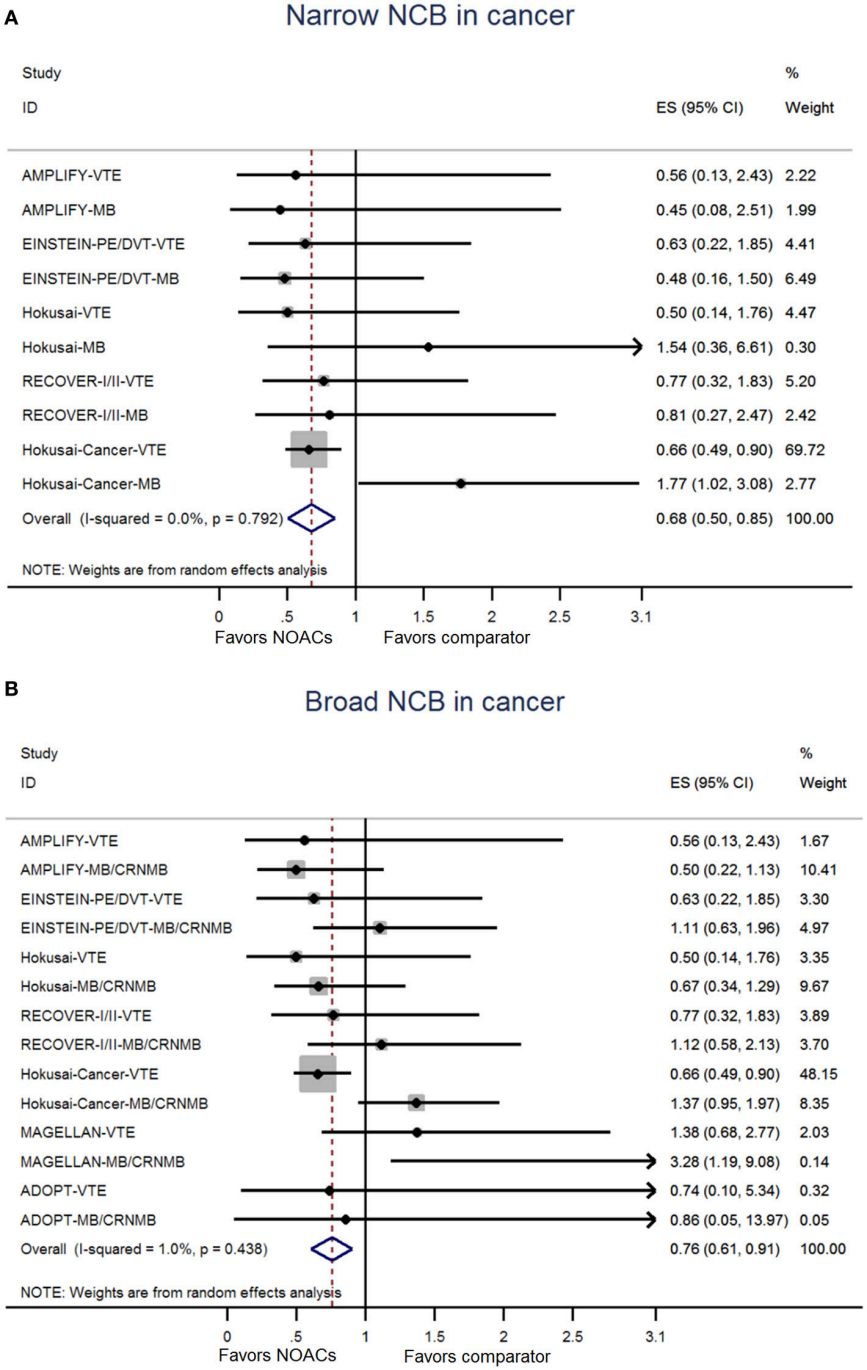
Analyses of narrow net clinical benefit **(A)** and broad net clinical benefit **(B)** in patients with cancer. ES indicates Odds ratio; 95%CI indicates 95% confidence interval.

**Figure 3:**
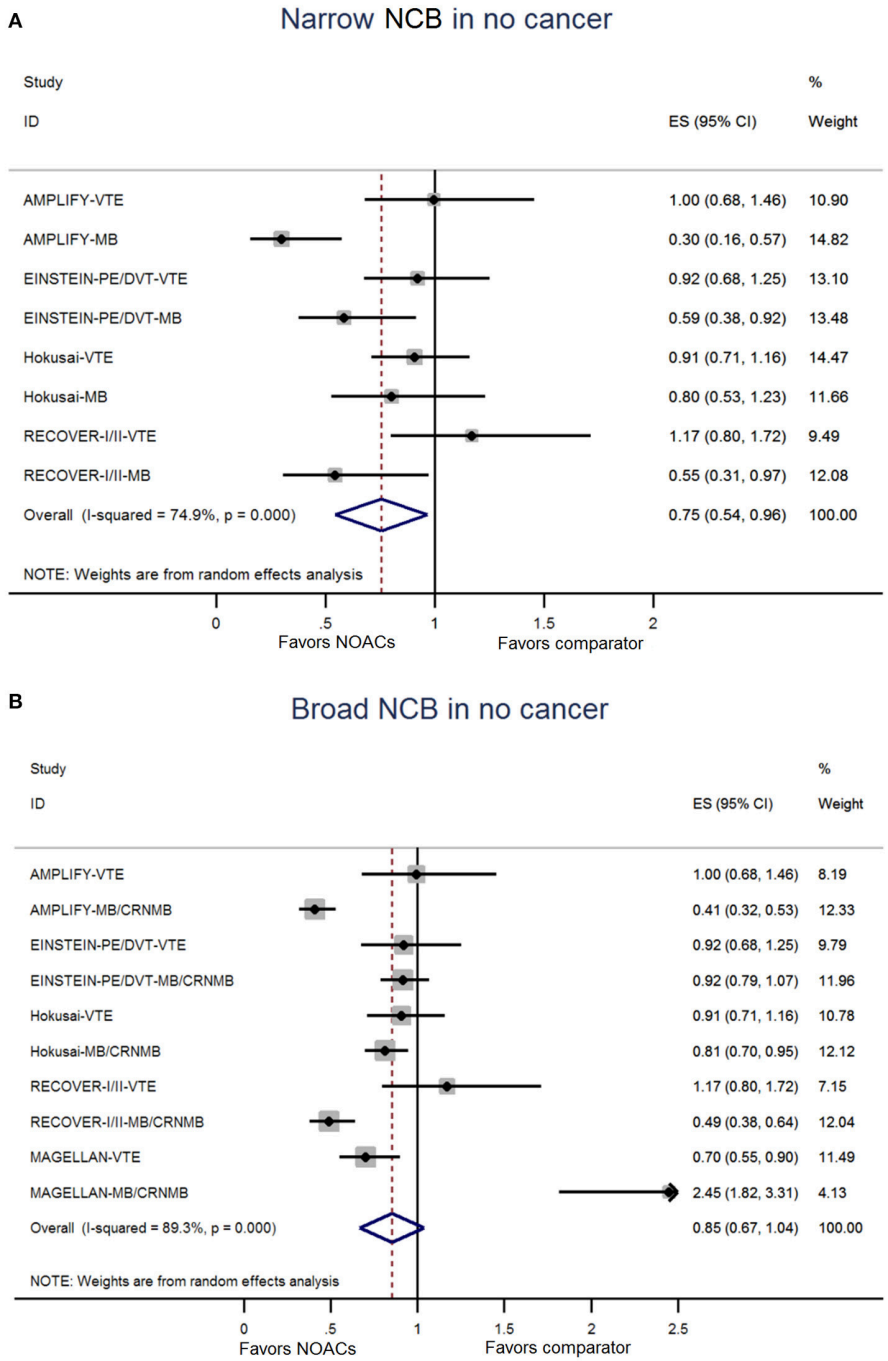
Analyses of narrow net clinical benefit **(A)** and broad net clinical benefit **(B)** in patients without cancer. ES indicates Odds ratio; 95%CI indicates 95% confidence interval.

The NCB analyses in patients with cancer were presented in Figures 2A,B. NOACs had a superior NCB than traditional anticoagulation, irrespective of narrow NCB (OR: 0.68, 95%CI: 0.50–0.85) and broad NCB (OR: 0.76, 95%CI: 0.61–0.91). No significant heterogeneity was detected in both narrow NCB analysis (*I*^2^: 0.0%; *P* = 0.792) and broad NCB analysis (*I*^2^: 1.0%; *P* = 0.438). In patients without cancer, as shown in Figures 3A,B, narrow NCB of NOACs was superior when compared with traditional anticoagulation (OR: 0.75, 95%CI: 0.54–0.96), and broad NCB showed a borderline significant result with NOACs vs. traditional anticoagulation (OR: 0.85, 95%CI: 0.67–1.04). The considerable heterogeneity was observed in narrow NCB analysis (*I*^2^: 74.9%; *P* < 0.01) as well as broad NCB analysis (*I*^2^: 89.3%; P < 0.01).

### NCB analysis based on different patients, comparators, and follow-up duration

NCBs analyses for subgroups were summarized in Tables 3, 4. As for VTE patients, the use of NOACs showed a positive broad NCB when compared to traditional anticoagulation in patients with cancer (OR: 0.76, 95%CI: 0.58–0.93) and without cancer (OR: 0.79, 95%CI: 0.61–0.98). No positive results were obtained in acutely ill patients due to limited sample size (OR: 1.39, 95%CI: 0.46–2.33 for broad NCB in cancer patients; OR: 1.54, 95%CI: 0.17–3.25 for broad NCB in no-cancer patients). In terms of different comparators, NOACs provided a better NCB compared with warfarin, regardless of patients with cancer (OR: 0.79, 95%CI: 0.61–0.98 for narrow NCB; OR: 0.71, 95%CI: 0.47–0.94 for broad NCB) and without cancer (OR: 0.79, 95%CI: 0.61–0.98 for broad NCB). However, these positive NCBs were not observed in comparison to LMWH (OR: 1.10, 95%CI: 0.03–2.17 for narrow NCB in cancer patients; OR: 1.05, 95%CI: 0.55–1.55 for broad NCB in cancer patients; OR: 1.54, 95%CI: 0.17–3.25 for broad NCB in no-cancer patients). With regard to follow-up duration, the results over 6 months showed that cancer patients treated with NOACs were associated with a better narrow NCB compared with traditional anticoagulation (OR: 0.68, 95%CI: 0.47–0.89). Consistent results were observed in patients without cancer (OR: 0.81, 95%CI: 0.65–0.97 for narrow NCB; OR: 0.87, 95%CI: 0.79–0.95 for broad NCB).

**Table 3:** Subgroup analyses for NCB in patients with cancer.

**Outcomes**	**No. of studies**	**OR**	**95%CI**	**Homogeneity**	
				***I*^2^ (%)**	***p*-value**
**NARROW NCB IN CANCER**					
**Class of control drugs**					
Warfarin	6	0.61	0.28–0.94	0.00	1.00
LMWH	1	1.10	0.03–2.17	76.9	0.04
**Follow up**					
≤ 6 months	3	0.68	0.19–1.18	0.00	0.96
>6 months	4	0.68	0.47–0.89	3.40	0.40
**BROAD NCB IN CANCER**					
**Patients**					
VTE patients	7	0.76	0.58–0.93	11.20	0.34
Acutely ill patients	2	1.39	0.46–2.33	0.00	0.77
**CLASS OF CONTROL DRUGS**					
Warfarin	6	0.71	0.49–0.90	0.00	0.82
LMWH	3	1.05	0.55–1.55	45.8	0.10
**Follow up**					
≤ 6 months	5	0.75	0.44–1.07	0.00	0.65
>6 months	4	0.82	0.55–1.08	37.80	0.15

*NCB, net clinical benefit; LMWH, low molecular weight heparin; VTE, venous thromboembolism; OR, odds ratios; CI, confidence intervals*.

**Table 4:** Subgroup analyses for NCB in patients without cancer.

**Outcomes**	**No. of studies**	**OR**	**95%CI**	**Homogeneity**	
				***I*^2^ (%)**	***p*-value**
**NARROW NCB IN NO CANCER**					
**Follow up**					
≤ 6 months	3	0.72	0.32–1.12	83.00	0.00
>6 months	3	0.81	0.65–0.97	23.80	0.27
**BROAD NCB IN NO CANCER**					
**Patients**					
VTE patients	6	0.79	0.61–0.98	88.80	0.0
Acutely ill patients	1	1.54	0.17–3.25	95.00	0.00
**CLASS OF CONTROL DRUGS**					
Warfarin	6	0.79	0.61–0.98	88.80	0.00
LMWH	1	1.54	0.17–3.25	95.00	0.00
**Follow up**					
≤ 6 months	4	0.85	0.58–1.12	89.40	0.00
> 6 months	3	0.87	0.79–0.95	0.00	0.69

*NCB, net clinical benefit; LMWH, low molecular weight heparin; VTE, venous thromboembolism; OR, odds ratios; CI, confidence intervals*.

### Sensitivity analyses and publication bias

Results of sensitivity analyses, including broad NCBs and narrow NCBs, were consistent with those of the primacy analyses (Table 5). Publication bias was not performed because of the limited study number of 9.

**Table 5:** Sensitivity analyses.

**Study omitted**	**OR**	**95%CI**
**NARROW NCB IN CANCER**		
AMPLIFY	0.68	0.51-0.86
EINSTEIN-PE/DVT	0.69	0.51-0.87
Hokusai	0.68	0.51–0.86
RECOVER-I/II	0.67	0.49–0.85
Hokusai-Cancer	0.61	0.28–0.94
**BROAD NCB IN CANCER**		
AMPLIFY	0.81	0.64–0.99
EINSTEIN-PE/DVT	0.76	0.58–0.94
Hokusai	0.82	0.62–1.02
RECOVER-I/II	0.77	0.58–0.95
Hokusai-Cancer	0.75	0.52–0.97
MAGELLAN	0.74	0.59–0.88
ADOPT	0.79	0.61–0.98
**NARROW NCB IN NO CANCER**		
AMPLIFY	0.80	0.64–0.97
EINSTEIN-PE/DVT	0.76	0.48–1.04
Hokusai	0.72	0.46–0.99
RECOVER-I/II	0.74	0.50–0.98
**BROAD NCB IN NO CANCER**		
AMPLIFY	0.89	0.71–1.08
EINSTEIN-PE/DVT	0.84	0.63–1.05
Hokusai	0.87	0.64–1.10
RECOVER-I/II	0.89	0.67–1.10
MAGELLAN	0.79	0.61–0.98

*OR, odds ratio; 95%CI, 95% confidence interval*.

## Discussion

NCB that incorporates both the risk of VTE and major bleeding provides a more quantitatively informed basis for the decision-making on the optimal anticoagulant therapy in patients with cancer. To the best of our knowledge, this is the largest sample size analysis to pool 2,902 cancer patients for evaluation of NCB of NOACs. Our results indicated that NCB of NOACs, in both cancer and no cancer patients, were superior to that of traditional anticoagulation, with the estimates mainly from VTE patients, warfarin-treated patients, and patients with long-term anticoagulation treatment (>6 months).

### NCB in patients with or without cancer

At present, NOACs have been shown to be as effective as and safer than traditional anticoagulation for VTE prevention in no-cancer patients. Thus, the use of NOACs are currently recommended for the treatment and prophylaxis of VTE by international clinical guideline (Kearon et al., 2016). Our trade-off analyses from pooling 9 RCTs showed that no-cancer patients treated with NOACs had a better NCB compared with patients treated with traditional anticoagulation.

Cancer has been associated with an increased risk of thromboembolic events, and the coexist of cancer and VTE confers significantly greater risk of this issue (Caine et al., 2002; Prandoni et al., 2002). In a pooled analysis of data from 38 study populations, the authors estimated the annual incidence rate of VTE to be between 0.5 and 20% depending on the cancer type, and found that cancers of the brain and pancreas were associated with the highest risk of VTE (Horsted et al., 2012). In a study from the USA, cancer patients with VTE were three times more likely to be hospitalized, with an additional seven hospital days relative to patients without VTE. This translated into significantly higher total healthcare costs (US$74,959 vs. US$41,691 per patient; *p* < 0.001) (Khorana et al., 2013). The use of anticoagulants is the standard treatment for the prevention of VTE in cancer patients, while an 8–10% annual bleeding events occurs during anticoagulation therapy (Brose and Lee, 2008). Hence, it is essential to balance the benefit and risk of anticoagulation therapy in this fragile population.

Prior meta-analysis studies that involved about 1,000 patients have addressed that NOACs seem to be as effective and safe as VKAs for the prevention of VTE in patients with cancer (Larsen et al., 2014; Van Der Hulle et al., 2014; Vedovati et al., 2015). In fact, at least 1500 patients should be analyzed in order to demonstrate a reduction in VTE from 3 to 5%. Afterward, Brunetti et al reported a consistent result even after pooling data with VKA and LMWH (Brunetti et al., 2017). The latest study by Di Minno et al, which separated data on patients with active cancer and cancer history, suggested a significantly lower risk of VTE and a non-significantly lower risk of major bleeding for the use of NOACs in patients with active cancer when compared to the use of VKAs (Di Minno et al., 2017). However, direct head-to-head comparison with LMWH is necessary before NOACs can be routinely appiled for cancer-associated VTE patients. Encouragingly, the latest Hokusai-Cancer trial, which included 1050 patients with predominantly advanced cancer and acute symptomatic or incidental VTE, showed that the use of edoxaban (Xa factor inhibitor) for up to 12 months was non-inferior to the use of LMWH in terms of the composite outcome of VTE or major bleeding (Raskob et al., 2018). When regarding efficacy, the rate of VTE was numerically lower with edoxaban than LMWH owing to the lower rate recurrent symptomatic deep-vein thrombus with edoxaban. While in terms of safety, the risk of major bleeding was significantly increased with edoxaban than with LMWH, which was mainly due to the higher risk of upper gastrointestinal bleeding with edoxaban (Raskob et al., 2018). Accordingly, treatment with NOACs may reduce the risk of VTE at the expense of increased risk of major bleeding. In the present study, we focused on a core issue, namely, net clinical benefit that weighted the benefit and risk, and analyzed it by pooling currently avaiable RCTs. The results found that the use of NOACs posed a better NCB than traditional anticoagulants in patients with cancer.

After further analysis based on subgroups, we recognized that the positive results of NCB was derived mainly from trials on NOACs vs. warfarin. For patients with warfarin therapy, time in therapeutic range (TTR) could reflect the anticoagulation effects. During the management of RCTs, it is recognized that TTR would be well controlled. Nevertheless, due to the frequent interactions with chemothrapeutic agents and immunosuppressive agents in anticancer therapy, TTR is hard to do well in practice. Compared to LMWH, NOACs showed non-inferior NCB in patients with cancer. The main contribution of the result came from recently published Hokusai-Cancer trial (Raskob et al., 2018). In addition, positive NCB with NOACs therapy in cancer patients was primarily derived from VTE patients on long-term treatment (>6 months). Patients with VTE are usually recommended to receive long-term anticoagulation therapy (Kearon et al., 2016), and the superior NCB of NOACs emerges during the course of treatment. As for acutely ill patients, sample size limited our ability to make a definitive conclusions and further assessment of NCB in these patients should be conducted.

### Clinical challenge and implication

Treatment and prophylaxis of cancer-associated VTE is challenging, and guidelines recommend treatment with LMWH due to lower rate of thrombosis and similar rate of bleeding when compared with VKAs (Farge et al., 2013; Lyman et al., 2015; Kearon et al., 2016). However, the use of LMWH is burdensome because it requires daily subcutaneous injecions, which may limit its long-term adoption. Given this clincial setting, NOACs may represent an alternative choice because of oral route of administration, predictable dose response, no need for laboratory monitoring, and greater flexibility during invasive procedures. All these adventages makes NOACs more appealing for long-term prevention of VTE. Undeniably, interactions between chemothrapeutic agents and immunosuppressive agents with NOACs are still possible, and drugs that interfere P-glycoprotein or CYP3A4 may impact the anticoagulation effect of NOACs (Voukalis et al., 2016). Thus, It is important to acknowledge that our results cannot be extrapolated to the cancer patients with the presence of potiental drug-drug interaction, but may only be applied to patients with similar characteristics included in the present analysis.

## Limitations

Several limitations need to be considered. Firstly, included RCTs were not especially designed to assess VTE and bleeding risk of NOACs in patients with cancer, with the expection of Hokusai-Cancer trial. Therefore, the difference in the baseline characteristics in patients allocated to NOACs and VKAs/LMWH could not be excluded. Secondly, the definition of outcomes was not same across included RCTs. However, as revealed by the values of *I*^2^, heterogeneity among the RCTs in patients with cancer was low in our random-effects model. Thirdly, no admitted methods are available to estimate the NCB in the field of VTE recently, thus we carried out a trade-off analysis between VTE and bleeding using a weighting of 1.0. Fourthly, individual NOACs analysis was not performed due to the limited studies. Fifthly, we have not get access to patient-level data in relation to the type, the stage or the location of cancer, making powerful subgroup analysis unavailable. Sixth, included studies have not addressed the impact of NOACs on different stakeholders including healthcare providers, users, and policymakers. Finally, drug-drug interaction information that impacts NOACs or VKAs were not addressed in included studies, which may lead certain bias. Accordingly, Further real-world studies are necessary to be conducted for the assessment of NOACs use in these special patients.

## Conclusions

The use of NOACs, compared with traditional anticoagulation, conferred a better net clinical benefit profile in patients with cancer. Hence, NOACs may represent an alternative choice in cancer patients who are expected long-term treatment of anticoagulation.

## Author contributions

Z-CG and L-WW are the guarantors of the entire manuscript. Y-DY and CZ contributed to the study conception and design; data acquisition, analysis, and interpretation; drafting of the manuscript; critical revision of the manuscript for important intellectual content; and final approval of the version to be published. LS, Y-JS, and X-YL contributed to the data acquisition, analysis, and interpretation.

### Conflict of interest statement

The authors declare that the research was conducted in the absence of any commercial or financial relationships that could be construed as a potential conflict of interest.
