# Supplementary material for: Net Clinical Benefit of Non-vitamin K Antagonist Oral Anticoagulants for Venous Thromboembolism Prophylaxis in Patients With Cancer: A Systematic Review and Trade-Off Analysis From 9 Randomized Controlled Trials
Source: Front Pharmacol. 2018 Jun 12;9:575. doi: 10.3389/fphar.2018.00575 (PMC6005885; doi:10.3389/fphar.2018.00575)
Supplement: Supplementary file 1 [file Table_1.DOCX]

**Supplemental table1. Definition of VTE events in included studies**

| Study | Definition of VTE events |
| --- | --- |
| AMPLIFY | Fatal or nonfatal pulmonary embolism or deep-vein thrombosis. |
| EINSTEIN-DVT/PE | Fatal or nonfatal pulmonary embolism or deep-vein thrombosis. |
| Hokusai | Fatal or nonfatal pulmonary embolism or deep-vein thrombosis. |
| RE-COVER-I/II | Symptomatic venous thromboembolism or death associated with venous thromboembolism. |
| Hokusai-Cancer | Symptomatic new deep-vein thrombosis or pulmonary embolism, incidental new deep-vein thrombosis or pulmonary embolism involving segmental or more proximal pulmonary arteries, or fatal pulmonary embolism or unexplained death for which pulmonary embolism could not be ruled out as the cause. |
| MAGELLAN | Asymptomatic proximal deep vein thrombosis, symptomatic proximal or distal deep-vein thrombosis, symptomatic nonfatal pulmonary embolism, or death related to venous thromboembolism. |
| ADOPT | Death related to venous thromboembolism (i.e., sudden death for which pulmonary embolism could not be excluded as a cause), fatal or nonfatal pulmonary embolism, symptomatic deep-vein thrombosis, or asymptomatic proximal-leg deep-vein thrombosis as detected with the use of systematic bilateral compression ultrasonography. |

VTE: venous thromboembolism.
